# Primary hyperparathyroidism presenting as acute hypercalcemic crisis: a case report

**DOI:** 10.1186/s40463-022-00600-x

**Published:** 2023-01-11

**Authors:** Gia Gill, Veena Agrawal, Paul Kerr

**Affiliations:** 1 Department of Otolaryngology-Head and Neck Surgery, Max Rady College of Medicine, University of Manitoba, GB421 - 820 Sherbrook Street, Winnipeg, MB R3A 1R9 Canada; 2 Section of Endocrinology & Metabolism, Department of Internal Medicine, Max Rady College of Medicine, University of Manitoba, 750 Bannatyne Ave, Winnipeg, MB R3E 0W2 Canada

**Keywords:** Hyperparathyroid, Hypercalcemia, Parathyroid adenoma, Atypical adenoma

## Abstract

**Background:**

Hyperparathyroid crisis, or “parathyroid storm” is a rare manifestation of primary hyperparathyroidism, characterized by sudden onset of symptomatic, severe hypercalcemia (> 3.5 mmol/L). Hemorrhage into a parathyroid adenoma has rarely been reported as an inciting or associated event. We present a case of hemorrhage into a longstanding adenoma presenting with acute onset of profound hypercalcemia and associated complications.

**Case presentation:**

A 60-year-old male presented to hospital with sudden onset of confusion, muscle weakness, and ataxia. Initial labs showed serum calcium 4.79 mmol/L, parathyroid hormone 2043 ng/L; creatinine 364 μmol/L. Review of the patient’s medical history indicated a 4-year history of recurrent nephrolithiasis, but no prior documented calcium levels. The hypercalcemia did not respond to 5 days of aggressive medical management with fluid resuscitation, denosumab and calcitonin, and later pamidronate and cinacalcet. He continued to deteriorate, requiring intubation and continuous renal replacement therapy. Imaging demonstrated 4.8 cm cystic right paratracheal mass; Technetium (Tc99m) Sestamibi scintigraphy was non-localizing. Urgent parathyroidectomy was completed, revealing a 5 × 3.3 × 1.8 cm hemorrhagic, atypical hypercellular parathyroid. Unfortunately, the patient died from complications from anticoagulation therapy for treatment of deep vein thrombosis 4 weeks after admission. His renal function had not recovered at the time of his death.

**Conclusion:**

This case gives potential insight into the etiology of hyperparathyroid crisis, and the difficulty in achieving control of hypercalcemia with medical means. Surgical intervention is the definitive management in these cases and should be considered urgently.

**Graphical Abstract:**

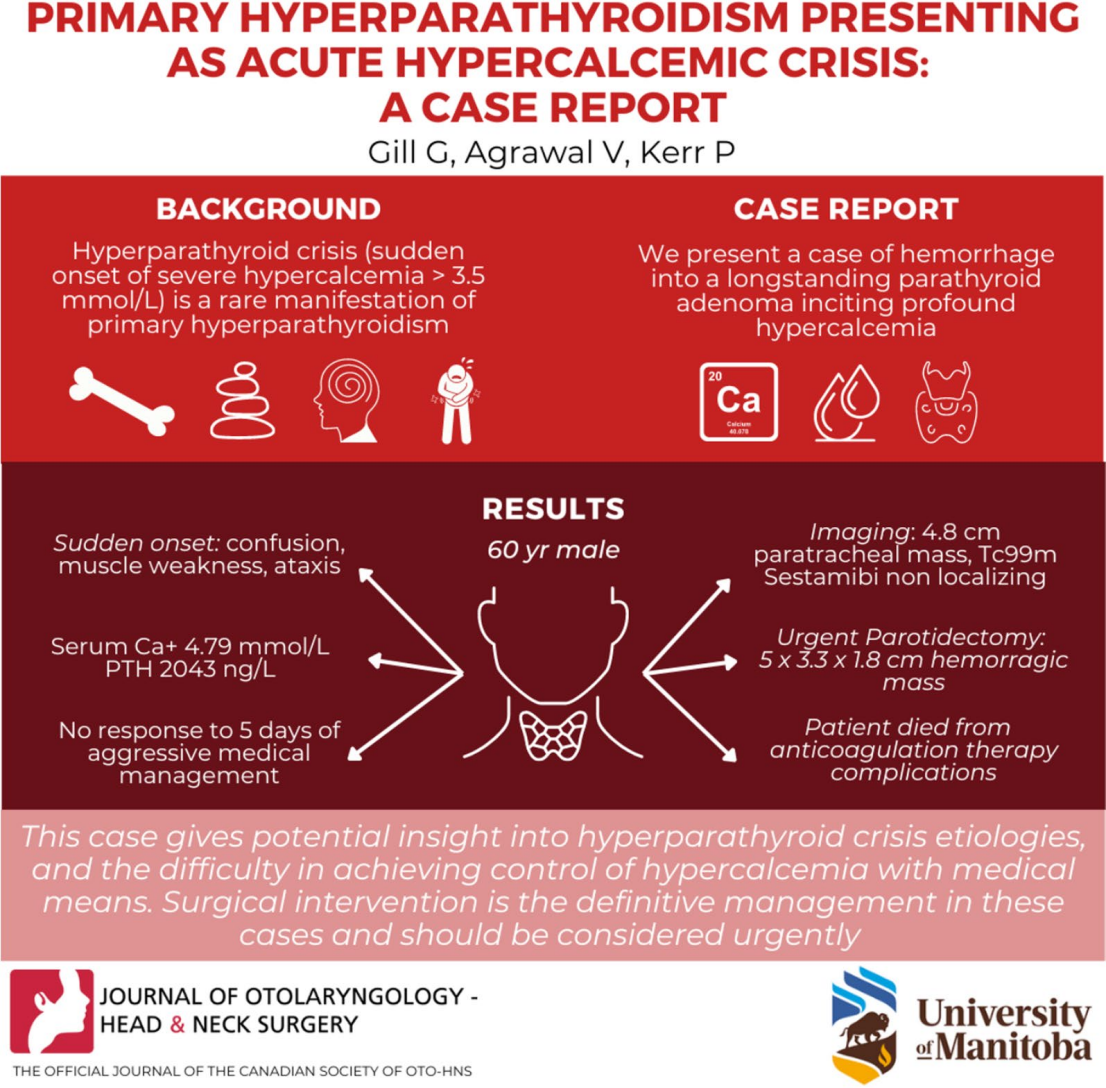

## Background

Primary hyperparathyroidism most commonly presents as an asymptomatic condition with mild hypercalcemia on biochemical screening [1, 2] On rare occasion, however, patients can present with dramatic symptoms related to sudden, severe hypercalcemia due to a rapid rise in parathyroid hormone (PTH). This hypercalcemic crisis, typically characterized by calcium levels > 3.5 mmol/L [3] has been referred to as hyperparathyroid crisis, or parathyroid storm, in the literature [4, 5]. The etiology of hyperparathyroid crisis is often unclear. This is a life-threatening condition requiring swift recognition and treatment, that may be relatively resistant to the usual medical management of hypercalcemia. Prompt surgical intervention should be considered if there is not an immediate response to medical management.

We report a case of parathyroid storm with the objective of illustrating some of the potential pathophysiologic mechanisms behind these cases, their relative resistance to medical management, and responsiveness to surgery.

## Case presentation

A 60-year old male with a history of recurrent nephrolithiasis requiring lithotripsy, gastroesophageal reflux disease, and mild chronic kidney disease presented to urgent care with a one-week history of rapidly increasing confusion, weakness, and gait abnormalities, with a preceding one-month history of headache and malaise. Further questioning also revealed nausea, decreased appetite, constipation and abdominal pain.

His initial bloodwork revealed an elevated corrected calcium of 4.79 mmol/L (normal 2.10–2.60 mmol/L), phosphate of 1.83 mmol/L (0.81–1.45 mmol/L), creatinine of 364 μmol/L (44–106 μmol/L; baseline creatinine ~ 130–150 µmol/L) with eGFR of 14.9 mL/min (> 60 mL/min), and markedly elevated PTH of 2043 ng/L (17–60 ng/L). Therefore, the diagnosis of severe primary hyperparathyroidism was established. Despite his history of recurrent nephrolithiasis, there was no previous documentation of serum calcium or parathyroid hormone levels in the patient’s provincial record.

**Fig. 1:**
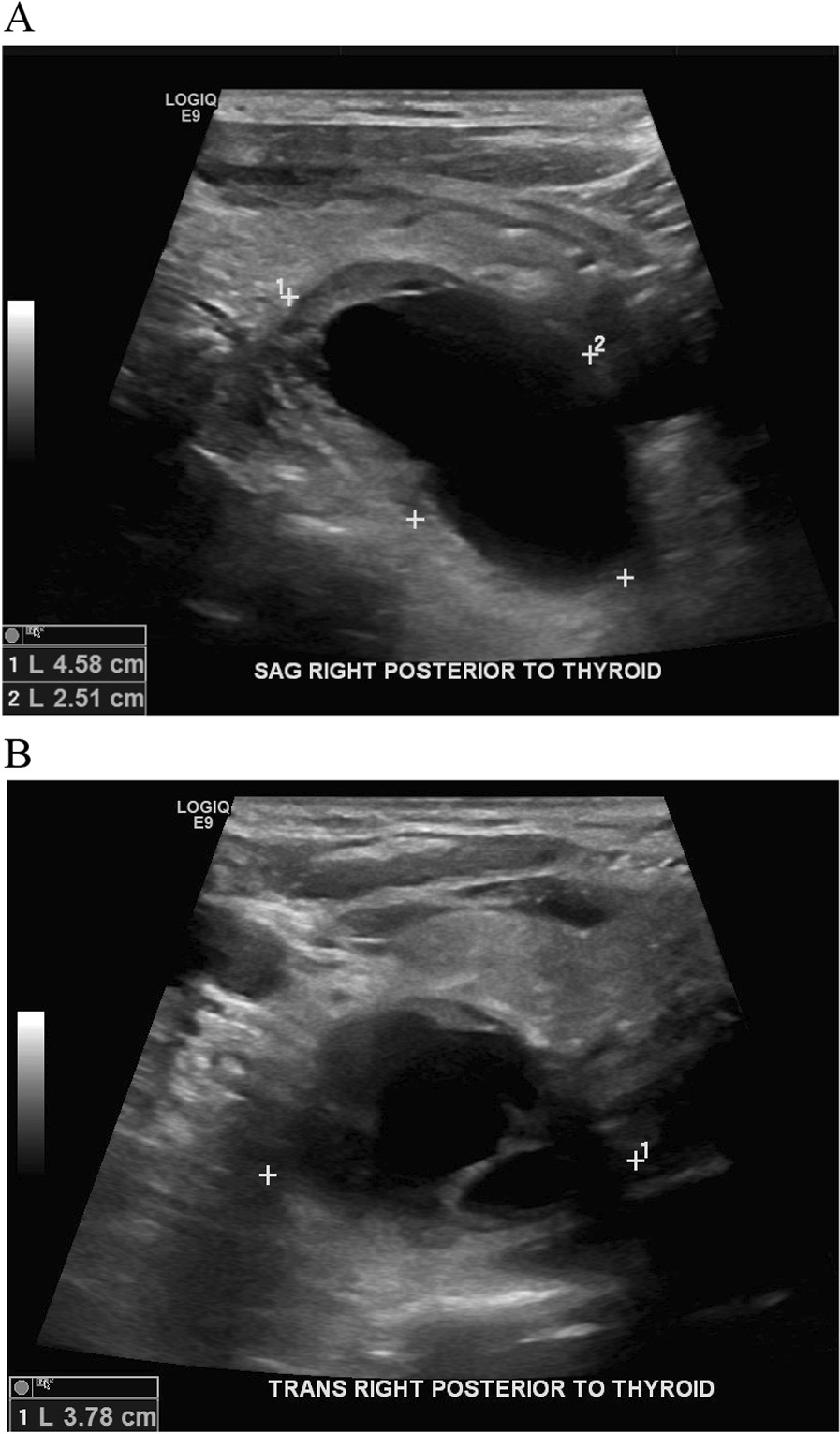
Ultrasound revealing mixed solid/cystic mass posterior to inferior right thyroid measuring 4.6 × 3.8 × 0.5 cm with prominent vascularity in solid rim surrounding cystic portion of mass. **A** Sagittal view **B** Transverse view

**Fig. 2:**
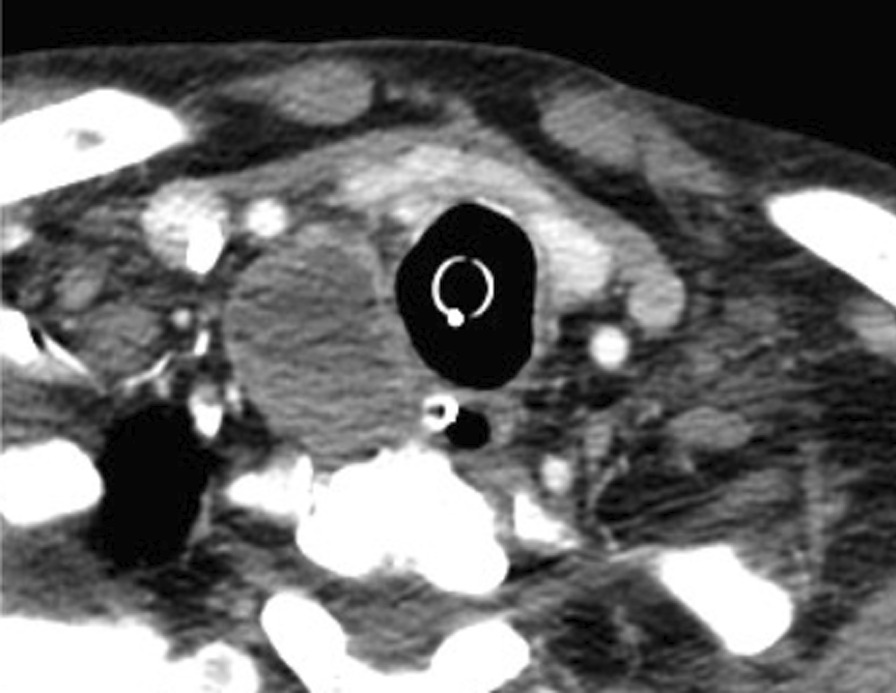
CT neck revealing right paratracheal neck mass

**Fig. 3:**
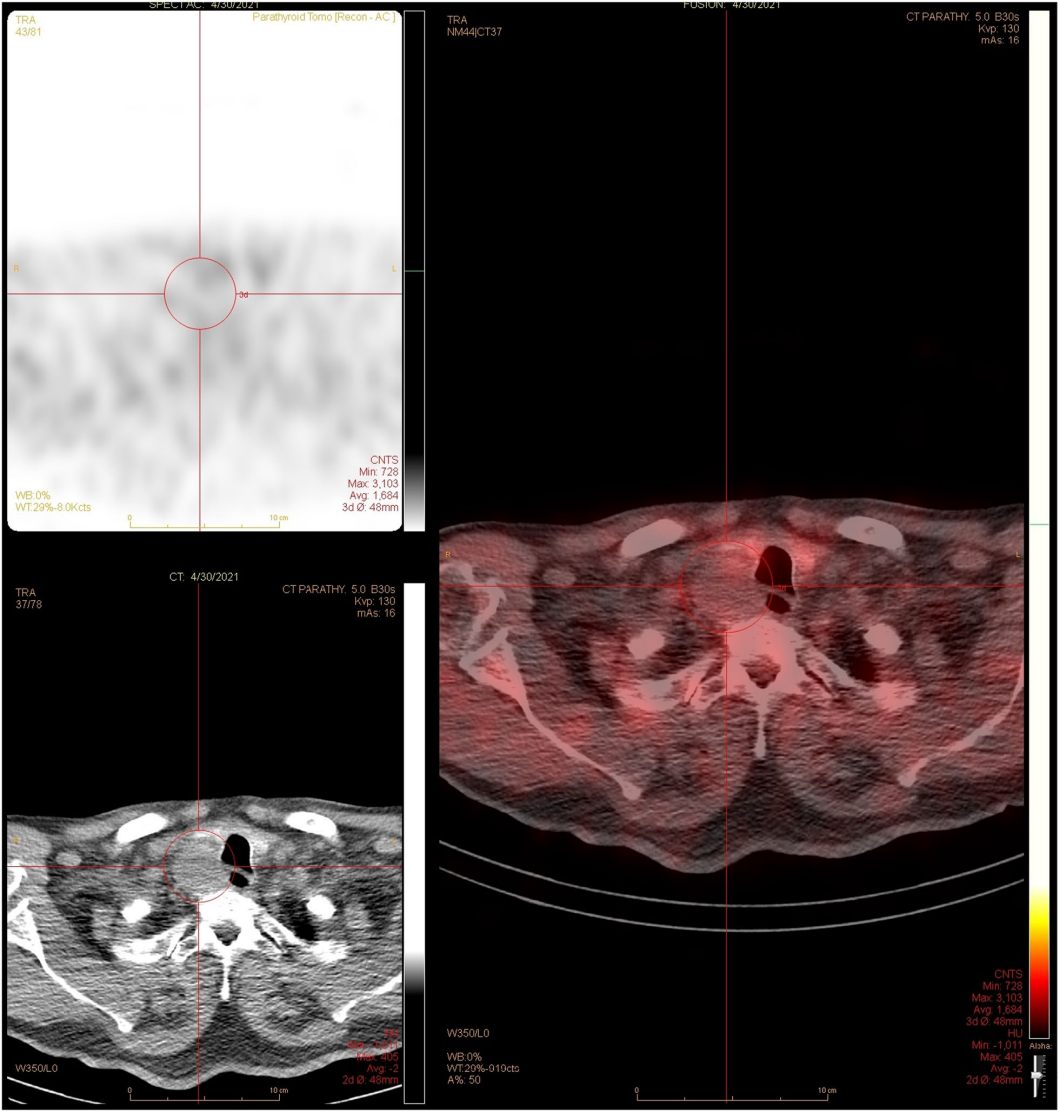
Dual tracer scan using 99 m T Sestamibi and 99 m Technetium, early and delayed phase

Ultrasound (US) showed a mixed solid and cystic mass located posterior to the right thyroid lobe measuring 4.6 × 3.8 × 2.5 cm in diameter, with prominent vascularity, raising suspicion for parathyroid carcinoma (Fig. 1 A and B). Computed tomography (CT) neck also described a 3.0 × 3.3 × 4.8 cm cystic, peripherally enhancing right paratracheal mass, with loss of soft tissue plane between the mass and adjacent tracheal tissue (Fig. 2). The technetium sestamibi scintigraphy was non-localizing and did not show increased uptake in the region of the neck mass seen on CT and US (Fig. 3).

The patient was admitted to the hospital under the Internal Medicine service and managed initially with aggressive fluid resuscitation, denosumab, and calcitonin. His calcium level continued to rise, with levels ranging from 5.14 to 5.26 mmol/L. His condition deteriorated, and he developed nephrogenic diabetes insipidus, hypernatremia, volume overload, and hypoxemia. He was transferred to intensive care 3 days post-admission for intubation, monitoring and continuous renal replacement therapy. Cinacalcet and pamidronate were added for hypercalcemia management.

**Fig. 4:**
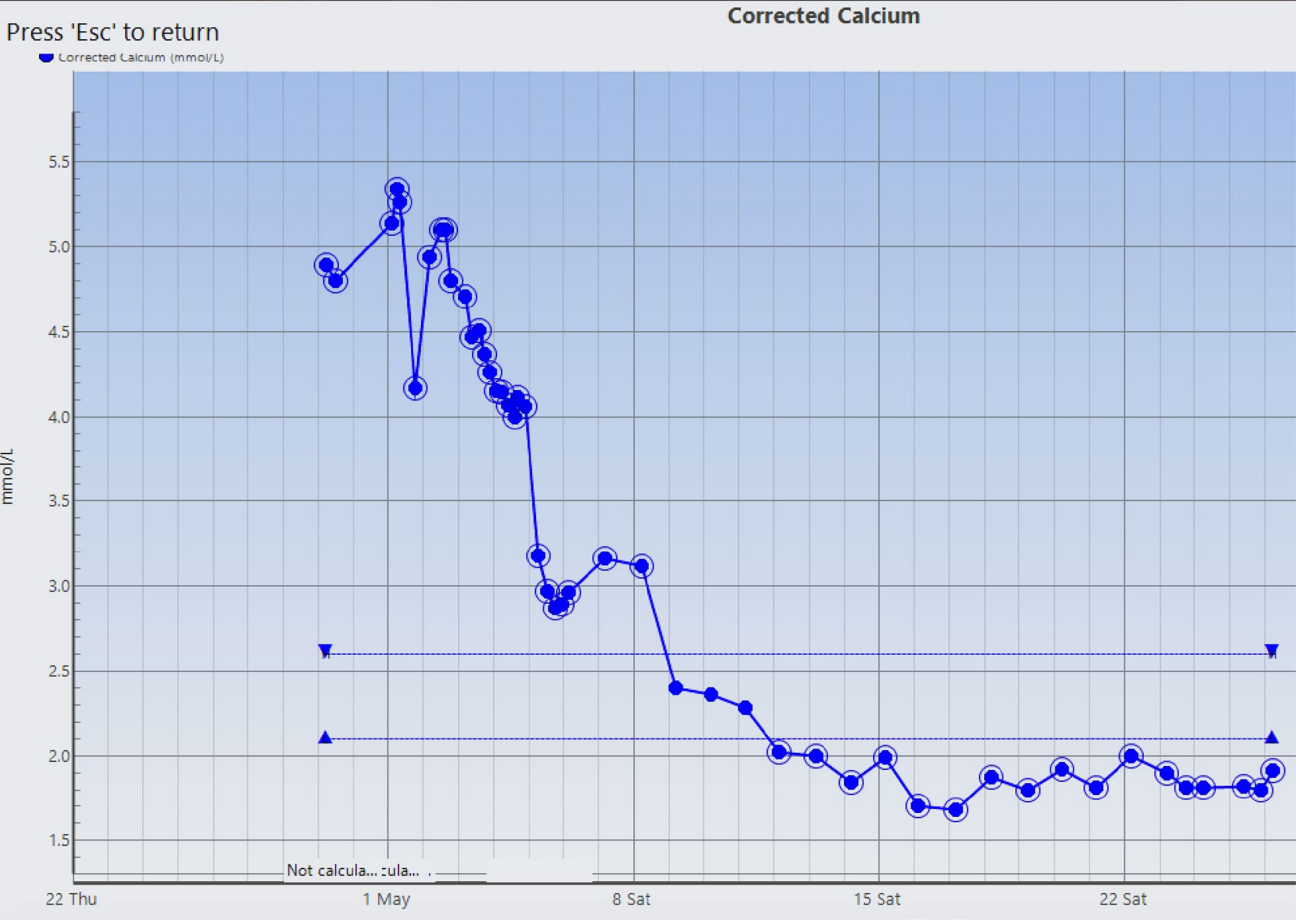
Trend of corrected calcium from presentation (28/04/21) until surgery (05/05/21)

**Fig. 5:**
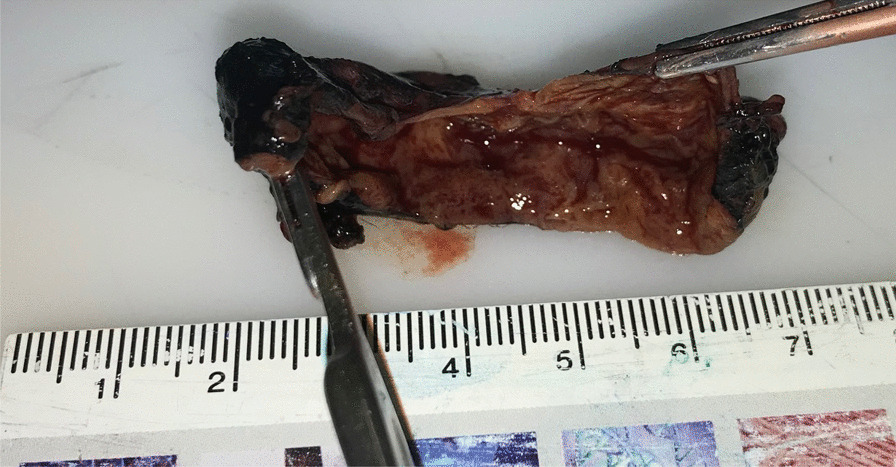
Specimen identified pathologically as right parathyroid adenoma

**Fig. 6:**
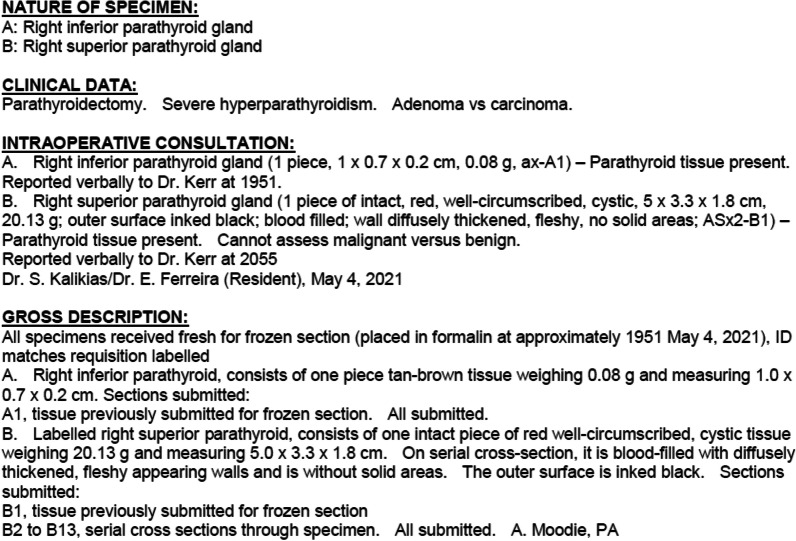
Pathology report for right inferior and superior parathyroid glands

The patient’s hypercalcemia did not adequately respond to maximal medical management (Fig. 4) so he underwent parathyroidectomy on post-admission day 5. Neck exploration revealed a fibrotic, inflamed parathyroid gland that was adherent to, but not invading, surrounding tissues. It was ultimately removed intact, without having to resect any of the surrounding structures. Pathologic examination described a 20 g parathyroid gland, measuring 5 × 3.3 × 1.8 cm. It was described as a blood-filled cyst. The gland was infarcted and surrounded by a fibrous, thickened capsule (Fig. 5). The final pathologic diagnosis was an atypical hypercellular parathyroid adenoma (Fig. 6), with capsular irregularities but no invasive features or cytologic atypia.

Calcium levels improved immediately following surgery, and calcium supplementation and alfacalcidol were eventually required. Unfortunately, the patient suffered a complicated course in hospital, with ongoing renal failure and development of atrial fibrillation, ventilator-associated pneumonia, and internal jugular venous thrombosis requiring heparin infusion. He ultimately died from complications related to his anticoagulation therapy.

## Discussion and conclusions

Our patient appears to have suffered hyperparathyroid crisis superimposed on long-standing primary hyperparathyroidism that had not been properly diagnosed at time of his initial presentation with renal calculi several years prior. The profoundly high PTH and calcium levels, rapid development of severe symptoms and signs, and pathology findings support the diagnosis of parathyroid storm from evolving hemorrhage and infarction within an atypical adenoma.

Hyperparathyroid crisis is a rare manifestation of primary hyperparathyroidism occurring in approximately 1% of patients [3, 6]. Although several studies have demonstrated that the average elevation in PTH and calcium is significantly higher in parathyroid carcinomas than in benign adenomas, the majority of patients with severe elevations in PTH and calcium still have benign disease [3, 6, 7]. Our patient had a PTH that was greater than 30 times the upper limit of normal, but pathology revealed only atypia.

The pathologic finding of atypical parathyroid adenoma is rare, and is likely a contributing factor in the degree of hyperparathyroidism seen in this case. Atypical parathyroid adenomas represent a neoplasm of uncertain malignant potential, accounting for only 1% of patients presenting with hyperparathyroidism [8]. A consensus on the exact number of histologic atypical features needed to make the diagnosis does not exist, though most studies require 2 of the following features: intraoperative adherence, bands of fibrosis, trabecular growth, necrosis, cellular atypia, sheet-like monotonous small cells with high nuclear/cytoplasmic ratio, and absence of unequivocal signs of malignancy [9–11]. A systematic review of 92 articles on atypical parathyroid adenomas described the biochemical profile of patients with atypical parathyroid adenoma as more similar to that of parathyroid carcinoma, with PTH levels with a mean of 12 times the upper limit of normal [8]. Galani et al. performed a retrospective cohort study on 117 patients with primary hyperparathyroidism, and found significantly higher preoperative serum calcium levels and symptomatic hypercalcemia in patients with atypical parathyroid adenoma when compared to benign adenoma [12].

The presence of hemorrhagic infarction may explain the sudden onset of further symptoms in our patient. First reported in 1934, spontaneous parathyroid hemorrhage has been associated with changes in parathyroid hormone secretion, as well as neck hematoma [5, 13, 14]. Hemorrhage causes damage to the gland, reportedly causing cystic degeneration and acute necrosis in a short time16 [15]. Previous studies have found significantly higher levels of serum PTH and calcium, as well as hypercalcemic crisis, in patients with cystic adenomas versus solid adenomas [16–18]. The pathophysiology of this release is due to the underlying changes seen in parathyroid adenomas. A review of 1754 patients with primary hyperparathyroidism revealed excessive number of intraparathyroid vacuoles containing high levels of PTH; cystic degeneration and lysis of these vacuoles could lead to substantial PTH release [4]. Large, cystic adenomas can mimic parathyroid carcinoma as seen in our case. Asghar et al. describe a similar presentation in a 55 year old female presenting in parathyroid crisis with PTH level of 1182 ng/L [19]. After minimal improvement with Calcitonin and Pamidronate, she underwent surgical excision of a 11 cm cystic parathyroid adenoma. Due to the impressive elevation of PTH there was suspicion of a possible parathyroid carcinoma. However, pathology revealed cystic degeneration of parathyroid adenoma. Interestingly, this patient also presented with internal jugular vein thrombosis which was proposed to be secondary to compression from the mass. This has been reported previously in the literature and may offer alternative etiology for thrombosis in our patient [20].

The cystic nature of the gland could also explain the negative technetium sestamibi scintigraphy found in our patient. A similar case to ours reported by Monsour et al. described a 69 year old female presenting with altered mental status and calcium level of 4.9 mmol/L [16]. Further workup revealed a PTH of 731 ng/L and a 3.5 cm left sided paratracheal mass. Parathyroid sestamibi scan was negative. The patient ultimately underwent left inferior parathyroidectomy, revealing an atypical cystic parathyroid adenoma. Larger studies have reported similar findings of negative scintigraphy in patients with cystic adenomas. Hu et al. reviewed 907 patients at their institution presenting with primary hyperparathyroidism [17]. Their study found significantly higher rates of negative technetium sestamibi scintigraphy in patients with cystic adenomas (19.4%), versus solid adenomas (4.3%). Johnson et al. reported preoperative localization in only 29% of patients with cystic adenomas [21].

Management of hypercalcemic crisis is frequently challenging. While bisphosphonates may shorten the time to normalization of calcium, patients frequently experience ongoing severe hypercalcemia despite traditional medical measures including intravenous fluids, bisphosphonates, and calcitonin [3, 22]. Guidelines from the Society for Endocrinology suggest the use of glucocorticoids, calcimemetics, denosumab, and calcitonin for refractory cases [23]. Calcimimetics such as cinacalcet act by allosteric activation of the calcium-sensing receptor, lowering calcium and plasma PTH concentrations [24]. Though typically indicated in secondary hyperparathyroidism, metanalysis has shown modest, temporary benefit in patients with moderate primary hyperparathyroidism [25]. Denosumab, a human monoclonal antibody, inhibits the protein RANKL and prevents osteoclast formation [26]. A randomized control trial has supported the use of denosumab in patients with primary hyperparathyroidism [27]. There is limited data on its efficacy in patients presenting with severe hypercalcemia, though case series suggest it may be considered in select cases [26]. These adjunct treatments are appropriate in patients who are unable to undergo surgical intervention, or to stabilize patients awaiting surgery.

In summary, hyperparathyroid crisis is a rare, life-threatening entity. Patients with recurrent nephrolithiasis should be screened for hyperparathyroidism with serum calcium and parathyroid hormone [28, 29]. Potential etiologies of acute hyperparathyroid crisis include malignancy, atypical adenoma, and hemorrhagic, cystic degeneration. Cystic parathyroid adenomas are associated with high serum levels of PTH and calcium, low accuracy of preoperative localization tests, and increased risk of hypercalcemia crisis [15–17]. These patients may warrant more urgent surgical intervention. The hypercalcemia of parathyroid storm often does not respond to maximal medical therapy, so urgent surgery should be considered early in the course of management.

## Data Availability

Data sharing is not applicable to this article as no datasets were generated or analysed during the current study.

## Abbreviations

PTH: Parathyroid hormone
US: Ultrasound
CT: Computed tomography
